# AI-Enhanced POCUS in Emergency Care

**DOI:** 10.3390/diagnostics16020353

**Published:** 2026-01-21

**Authors:** Monica Puticiu, Diana Cimpoesu, Florica Pop, Irina Ciumanghel, Luciana Teodora Rotaru, Bogdan Oprita, Mihai Alexandru Butoi, Vlad Ionut Belghiru, Raluca Mihaela Tat, Adela Golea

**Affiliations:** 1 Faculty of Medicine, Vasile Goldis Western University of Arad, 310025 Arad, Romania; puticiu.monica@uvvg.ro (M.P.); pop.florica@uvvg.ro (F.P.); 2 Faculty of Medicine, “Grigore T. Popa” University of Medicine and Pharmacy, 700115 Iasi, Romania; carmen.cimpoesu@umfiasi.ro; 3 Emergency Department, Emergency “St. Spiridon” Hospital, 700111 Iasi, Romania; 4 Emergency Medicine and First Aid Department, Faculty of Medicine, University of Medicine and Pharmacy, 200349 Craiova, Romania; mihai.butoi@rmu.smurd.ro (M.A.B.); vlad.belghiru@yahoo.com (V.I.B.); 5 Faculty of Medicine, “Carol Davila” University of Medicine and Pharmacy, 050474 Bucharest, Romania; bogdan.oprita@umfcd.ro; 6 Department of Emergency Medicine, Clinical Emergency Hospital of Bucharest, 014461 Bucharest, Romania; 7 Department 6 Surgery, Emergency Medicine Discipline, “Iuliu-Hațieganu” University of Medicine and Pharmacy, 400012 Cluj-Napoca, Romania; raluca.tat@umfcluj.ro (R.M.T.); adela.golea@umfcluj.ro (A.G.)

**Keywords:** artificial intelligence, point-of-care ultrasound, POCUS, emergency medicine, diagnostic imaging, machine learning, deep learning

## Abstract

Point-of-care ultrasound (POCUS) is an essential component of emergency medicine, enabling rapid bedside assessment across a wide spectrum of acute conditions. Its effectiveness, however, remains constrained by operator dependency, variable image quality, and time-critical decision-making. Recent advances in artificial intelligence (AI) offer opportunities to augment POCUS by supporting image acquisition, interpretation, and quantitative analysis. This narrative review synthesizes current evidence on AI-enhanced POCUS applications in emergency care, encompassing trauma, non-traumatic emergencies, integrated workflows, resource-limited settings, and education and training. Across trauma settings, AI-assisted POCUS has demonstrated promising performance for automated detection of pneumothorax, hemothorax, and free intraperitoneal fluid, supporting standardized eFAST examinations and rapid triage. In non-traumatic emergencies, AI-enabled cardiovascular, pulmonary, and abdominal applications provide automated measurements and pattern recognition that can approach expert-level performance when image quality is adequate. Integrated AI–POCUS systems and educational tools further highlight the potential to expand ultrasound access, support non-expert users, and standardize training. Nevertheless, important limitations persist, including limited generalizability, dataset bias, device heterogeneity, and uncertain impact on clinical decision-making and patient outcomes. In conclusion, AI-enhanced POCUS is transitioning from proof-of-concept toward early clinical integration in emergency medicine. While current evidence supports its role as a decision-support tool that may enhance consistency and efficiency, widespread adoption will require prospective multicentre validation, development of representative POCUS-specific datasets, vendor-agnostic solutions, and alignment with clinical, ethical, and regulatory frameworks.

## 1. Introduction

Point-of-care ultrasound (POCUS) has rapidly become an indispensable diagnostic and clinical tool across a broad spectrum of healthcare settings, enabling clinicians to perform real-time, bedside imaging that directly informs patient care. Traditionally, the utility of POCUS has been limited by operator dependency and variability in interpretation, highlighting the need for solutions that can augment clinician performance and standardize results.

In the context of medical imaging and point-of-care ultrasound (POCUS), artificial intelligence (AI) encompasses computational methods designed to support image analysis and clinical decision-making [1]. Early applications relied on rule-based systems using predefined criteria, whereas most contemporary approaches are based on machine learning, in which algorithms learn relevant patterns from data. Deep learning, a subset of machine learning, employs multilayer neural networks—most commonly convolutional neural networks—to automatically extract features from ultrasound images and video clips [2]. In this review, the term “AI-enhanced POCUS” primarily refers to machine learning– and deep learning–based systems that assist with image acquisition, quality assessment, and interpretation, while final clinical decisions remain clinician-driven.

The development of AI-enhanced POCUS parallels earlier adoption of artificial intelligence in adjacent medical fields such as radiology, echocardiography, digital pathology, and clinical decision support systems [3,4,5]. In radiology and pathology, AI has demonstrated robust performance in image classification, segmentation, and workflow prioritization, largely benefiting from standardized acquisition protocols and high-quality datasets [6,7]. Similarly, in echocardiography, AI-based tools for automated chamber quantification and ejection fraction estimation are increasingly integrated into clinical practice [8]. In contrast, POCUS presents unique challenges related to operator dependency, heterogeneous devices, and variable imaging conditions [9]. These differences underscore both the novelty of AI–POCUS applications and the need to adapt established AI concepts to the constraints of real-time, bedside imaging, while also supporting the transferability of validated methodologies across imaging domains.

Emergency medicine ultrasound is the bedside use of POCUS by emergency physicians to quickly evaluate and manage patients with acute conditions. In practice, POCUS has become closely linked to the clinical exam, helping physicians combine their usual assessment skills with real-time imaging during patient care. Emergency medicine ultrasound applications are often grouped by anatomical region or by clinical purpose—such as resuscitation, diagnosis, procedural guidance, or symptom-based assessments. The way these applications are used varies considerably between regions and health systems, depending on available training, equipment, and local priorities [1]. The predominant use of AI involved automated biometric measurements derived from POCUS images, particularly in acute and cardiopulmonary settings, improving accessibility, streamlining workflows, and showing promising diagnostic accuracy for specific tasks such as ejection fraction estimation and identification of B-lines in lung imaging, while also underscoring limitations in image quality and generalizability of models [2]. AI-POCUS research increasingly targets low-resource environments, particularly ow- and middle-income countries, rural or remote regions, and emergency settings [9].

The integration of AI in POCUS is not without challenges. Clinicians report enthusiasm for AI assistance but also highlight barriers including training and education gaps, the need for robust clinical validation, workflow integration issues, and ethical/regulatory concerns [10]. Additionally, standardization of protocols, device interoperability, and algorithm transparency remain pressing obstacles to widespread adoption. Despite these hurdles, emerging evidence suggests that AI-augmented POCUS could democratize access to advanced imaging in both high-resource and low-resource environments, with applications ranging from automated anatomical landmark detection to real-time feedback for novice users [9].

Despite its promising potential, the integration of artificial intelligence into point-of-care ultrasound also raises important risks and limitations. Algorithmic bias related to non-representative training datasets, variability in device hardware, and patient populations may compromise generalizability across clinical settings [11]. Overreliance on automated interpretation carries the risk of deskilling and inappropriate clinical decision-making, particularly in time-critical emergency contexts. In addition, unresolved medico-legal and regulatory questions—including accountability for AI-assisted diagnostic errors and transparency of algorithmic decision processes—remain significant barriers to widespread adoption [12]. These considerations underscore the need to position AI-enhanced POCUS as a decision-support tool that augments, rather than replaces, clinician expertise.

Accordingly, this narrative review aims to address the following research question: how is artificial intelligence currently being applied to point-of-care ultrasound in emergency care, and what evidence exists regarding its performance, clinical feasibility, and implementation challenges across different emergency settings? Specifically, we synthesize current data on AI-assisted POCUS applications in trauma and non-traumatic emergencies, integrated and resource-limited workflows, and education and training, with the goal of evaluating the translational readiness and future clinical role of AI-enhanced POCUS.

## 2. Materials and Methods

This narrative review was conducted using a structured literature search strategy informed by PRISMA reporting principles, adapted to the scope and objectives of a narrative synthesis. The aim was to ensure transparency and reproducibility in study identification, screening, and selection.

A comprehensive search was performed in the Web of Science (WoS) Core Collection and PubMed/MEDLINE databases to identify peer-reviewed articles published in English between 1 January 2020 and 1 December 2025. Web of Science was selected for its broad multidisciplinary coverage and citation indexing, while PubMed/MEDLINE was included to ensure comprehensive retrieval of clinically relevant biomedical literature.

The search strategy combined terms related to artificial intelligence, point-of-care ultrasound, and emergency care, including variations of “artificial intelligence,” “machine learning,” “deep learning,” “point-of-care ultrasound,” “POCUS,” and “emergency medicine.”

Inclusion criteria were as follows:

(i) Studies explicitly addressing the application of artificial intelligence in point-of-care ultrasound;
(ii) Relevance to emergency medicine or acute care settings;
(iii) Original research articles, technical development studies, clinical validation studies, or narrative/scoping reviews.

Exclusion criteria were as follows:

(i) Case reports or small case series;
(ii) Publications in languages other than English;
(iii) Studies not specifically involving AI-based applications in POCUS.

A total of 93 studies were included in the qualitative synthesis, comprising 38 articles identified from Web of Science and 55 from PubMed/MEDLINE, after screening and application of inclusion and exclusion criteria. Studies were grouped according to their primary clinical application domain: trauma assessment (6 articles), cardiovascular evaluation (10 articles), pulmonary imaging (12 articles), abdominal assessment (4 articles), and education and training (8 articles).

Most studies originated from North America and Europe, with fewer from Asia and low- and middle-income countries. Convolutional neural networks (CNNs) were the most frequently used AI models, followed by classical machine learning approaches and, less commonly, recurrent neural networks. Reported diagnostic performance metrics varied across applications, with most studies reporting AUC values between 0.85 and >0.90 and sensitivities and specificities typically in the 80–95% range. This heterogeneity reflects differences in datasets, validation strategies, and clinical endpoints, underscoring the importance of cautious interpretation and the need for standardized reporting in future studies.

Data extraction was performed using a standardized approach, focusing on study characteristics (design, clinical setting, POCUS application, AI methodology), primary objectives, and reported performance or feasibility outcomes. Methodological quality and reliability were assessed qualitatively, considering factors such as study design, dataset size, validation strategy, and clinical relevance. Given the heterogeneity of AI techniques, clinical applications, and outcome measures, a formal risk-of-bias or quality scoring tool was not applied, and findings were synthesized narratively with emphasis on consistency, limitations, and generalizability across studies.

## 3. Trauma and AI-Enhanced POCUS

The integration of artificial intelligence (AI) into point-of-care ultrasound (POCUS) is increasingly influencing the assessment of medical emergencies, particularly in trauma care where rapid and accurate decision-making is essential. In this context, AI systems have been proposed as tools to enhance diagnostic accuracy, standardize image interpretation, and reduce dependence on operator expertise.

Recent research has focused on the application of AI to support rapid evaluation of traumatic injuries using POCUS, especially within extended Focused Assessment with Sonography for Trauma (eFAST) examinations and thoracic imaging. Deep learning–based models have been developed for real-time interpretation of thoracic ultrasound images to identify pneumothorax and hemothorax. Experimental studies using convolutional neural network architectures have demonstrated meaningful classification performance, even when real-time accuracy was lower than that observed during model training, suggesting that AI assistance may lower the expertise threshold required for trauma triage in prehospital or resource-limited environments (Table 1).

Beyond diagnostic classification, AI systems have also been designed to support automated identification of key eFAST anatomical landmarks and to provide probe-positioning guidance, thereby assisting both image acquisition and interpretation. When combined with diagnostic classifiers, such guidance models have shown feasibility for real-time use in experimental settings and may help streamline rapid triage decisions in acute trauma scenarios [13].

In lung ultrasound, AI frameworks that mirror the clinical workflow—incorporating initial transducer placement, automated quality assessment, and dynamic evaluation of lung sliding—have achieved high diagnostic performance for pneumothorax detection. Integrated pipelines combining quality assurance with lung sliding classification have reported area under the receiver operating characteristic curve values approaching 0.89, supporting the potential of AI to assist less experienced users in urgent trauma settings [14].

Systematic investigations further indicate that AI-based ultrasound systems can achieve sensitivities in the mid-80% range for pneumothorax detection, even when trained on relatively limited datasets. Collectively, these findings suggest that automated pattern recognition in trauma POCUS can approach expert-level diagnostic performance and may expand the utility of ultrasound in emergency and trauma workflows [15].

## 4. Non-Traumatic Emergencies and AI-Enhanced POCUS

Before detailing organ-specific applications, the development of AI-enhanced POCUS should be viewed within the broader context of artificial intelligence applications across ultrasonography. In established domains such as echocardiography and obstetric ultrasound, AI tools for automated measurements and pattern recognition benefit from standardized acquisition protocols and large, curated datasets. Similarly, in radiology, automated quality assurance and workflow optimization systems are increasingly embedded in routine imaging practice. In contrast, POCUS operates under more variable conditions, with heterogeneous devices, operator-dependent image acquisition, and lower or inconsistent image quality. These differences highlight why, despite methodological overlap, AI–POCUS faces distinct translational challenges that warrant focused evaluation.

### 4.1. Cardiovascular Assessment

Cardiac point-of-care ultrasound (POCUS) is integral to the evaluation of non-traumatic emergencies such as acute dyspnea, chest pain, hypotension, and undifferentiated shock. In routine emergency practice, however, assessment of ventricular function and hemodynamic status remains highly operator-dependent and is often limited to qualitative visual estimation rather than formal quantitative measurements during time-critical encounters. Recent studies evaluating automated AI-based tools integrated into POCUS platforms—including automated ejection fraction (EF), velocity–time integral (VTI), and inferior vena cava (IVC) assessment—have demonstrated moderate to good agreement with expert interpretation, particularly when image quality is adequate. Notably, VTI estimation appears relatively robust even in medium-quality recordings, supporting the feasibility of embedding real-time quantitative AI assistance into emergency workflows without substantially increasing acquisition time [19].

Beyond single-centre validation studies, prospective emergency department data increasingly support the diagnostic accuracy of AI-enabled cardiac POCUS. In cohorts of emergency patients with risk factors for cardiac dysfunction, vendor-integrated AI software has demonstrated high sensitivity and specificity for the automated detection of both systolic and diastolic left ventricular dysfunction when compared with expert interpretation, provided that image quality is adequate. These findings indicate that AI-assisted analysis can approach expert-level classification of cardiac function in real-world ED settings. In parallel, fully automated wall-tracking approaches have been developed to estimate ejection fraction from parasternal long-axis views, which are often easier to acquire than apical views in critically ill patients. Such methods have shown accurate identification of clinically relevant ejection fraction thresholds, with performance comparable to apical-view assessments and superior to traditional parasternal techniques. Together with device-level solutions such as automated EF and velocity–time integral tools integrated into contemporary POCUS platforms, these studies suggest that automated quantification of ventricular function, stroke-volume surrogates, and inferior vena cava dynamics has reached a level of technical maturity compatible with routine emergency workflows [20].

Beyond global functional assessment, AI is increasingly applied to extend cardiac POCUS toward disease-specific screening. Recent studies have demonstrated that AI models applied to single-view cardiac POCUS can accurately identify conditions such as hypertrophic and transthyretin amyloid cardiomyopathy, with high discriminative performance across large health systems. Importantly, AI-based risk stratification enabled detection of cardiomyopathy years before clinical diagnosis and was independently associated with mortality, highlighting the potential of AI-enhanced POCUS as a scalable tool for early disease screening in emergency and acute care settings [21].

Neonatal and pediatric non-traumatic emergencies represent another emerging domain for AI-supported echocardiography. Targeted neonatal echocardiography and POCUS have become essential bedside tools for real-time hemodynamic assessment in neonatal intensive care units. In this context, machine learning and AI are increasingly viewed as promising adjuncts for rapid, physiology-driven decision-making, particularly where access to specialized pediatric cardiology expertise is limited. Although most neonatal AI applications remain conceptual or early-stage, this work situates AI–POCUS within a broader shift toward continuous and individualized hemodynamic monitoring in vulnerable populations [22].

At the same time, several studies underscore that AI performance is highly sensitive to data domain and image characteristics. Models trained on standard echocardiography have shown reduced performance when applied to emergency department POCUS images, with only moderate segmentation accuracy and poor agreement for ejection fraction classification, emphasizing the need for models trained specifically on POCUS datasets [23]. In settings with limited POCUS data, data augmentation strategies—such as view rotation and flipping—have partially improved performance when adapting models to subxiphoid views, although agreement remains weakest for mid-range ejection fraction values [24].

Beyond individual algorithms, broader analyses highlight persistent barriers to the clinical deployment of AI in POCUS, including concerns related to trustworthiness, bias, data governance, workflow integration, and the gap between proof-of-concept systems and regulated clinical tools [25]. These challenges are particularly pronounced in emergency settings, where image quality is often suboptimal, patient physiology is rapidly evolving, and opaque “black-box” outputs may limit clinician acceptance. Collectively, the available evidence supports prioritizing multicentre prospective validation in real-world emergency workflows, transparent reporting of failure modes, and the development of open, POCUS-specific training datasets that capture diverse patient populations and ultrasound platforms.

Table 2 summarizes the main studies evaluating artificial intelligence–enhanced POCUS for cardiovascular assessment in non-traumatic emergency settings, highlighting clinical context, AI applications, and key performance outcomes.

### 4.2. Lung Assessment

Point-of-care ultrasound has become a cornerstone of pulmonary evaluation in emergency and acute care due to its real-time imaging capabilities, absence of ionizing radiation, and high sensitivity for common conditions such as pneumothorax, pulmonary edema, pleural effusion, and interstitial syndrome. However, conventional lung ultrasound interpretation remains highly operator-dependent, particularly when identifying subtle artefacts and integrating dynamic findings into time-critical clinical decisions. The integration of artificial intelligence aims to standardize lung POCUS interpretation, automate detection of key sonographic signs, and support clinicians in high-acuity scenarios.

AI-enabled lung POCUS frameworks have been developed for real-time detection of pneumothorax and other thoracic pathologies, achieving diagnostic performance potentially suitable for emergency triage applications. Early systems have simulated clinical workflows by combining deep learning–based classifiers for pleural sliding with automated quality assurance, demonstrating the feasibility of AI-supported bedside assessment [14]. Building on this foundational work, subsequent models trained on large, annotated ultrasound video datasets have achieved high sensitivity and specificity for pneumothorax detection, with area under the receiver operating characteristic curves exceeding 0.90 when compared with expert interpretation. These findings highlight the potential clinical utility of AI-augmented lung POCUS for rapid triage of patients with suspected pleural air [30].

Beyond diagnostic classification, AI approaches have also been applied to the quantitative assessment of pulmonary congestion. Machine learning pipelines trained to identify, localize, and count B-lines across standard lung zones have shown strong correlation with expert annotations and established biomarkers of congestion, such as NT-proBNP. Automated B-line quantification may therefore provide objective metrics to guide decongestive therapy and monitor treatment response in acute heart failure presentations [31].

AI techniques have additionally been explored for the characterization of pleural effusions. Pattern recognition–based models have demonstrated improved differentiation of anechoic and complex pleural collections compared with unaided visual interpretation, particularly in cases with subtle septations or mixed echogenicity. This supports the role of AI as an adjunct for pleural pathology assessment and for procedural planning, including ultrasound-guided thoracentesis [32].

Finally, preliminary studies have reported early integration of AI into lung POCUS workflows with a focus on standardization and training-oriented feedback. Prototype systems combining AI-based image analysis with real-time operator guidance have been described, automatically suggesting adjustments in probe orientation and scanning technique to improve acquisition quality. These approaches align with broader goals of AI-supported POCUS, in which artificial intelligence contributes not only to image interpretation but also to optimization of image acquisition at the bedside [33].

Key studies investigating AI-enhanced lung POCUS in non-traumatic emergencies, including clinical applications, AI methodologies, and reported diagnostic performance, are summarized in Table 3.

### 4.3. Abdominal Assessment

Abdominal point-of-care ultrasound plays an important role in the evaluation of non-traumatic emergencies, particularly in patients presenting with acute abdominal pain, suspected intra-abdominal bleeding, bowel obstruction, urinary retention, or gynecologic pathology. Although abdominal POCUS is widely used to identify findings such as free intraperitoneal fluid, hydronephrosis, gallbladder disease, or bladder distension, diagnostic accuracy remains strongly dependent on operator experience and image interpretation skills. Artificial intelligence has therefore been explored as a means to support image acquisition, automate detection of key sonographic findings, and improve diagnostic consistency during time-sensitive abdominal assessments.

Recent studies have examined AI-assisted ultrasound applications in acute obstetric and gynecologic emergencies, focusing on machine learning–based algorithms that facilitate real-time differentiation between intrauterine and ectopic pregnancy and enable rapid risk stratification in cases of early pregnancy bleeding. These approaches suggest that AI-enhanced abdominal and pelvic POCUS may promote more standardized interpretation and faster clinical decision-making in emergency settings where access to expert sonographers or comprehensive imaging is limited, although further prospective validation is required [42].

Complementary exploratory work has investigated deep learning methods for automated recognition of free intraperitoneal fluid and abdominal organ boundaries using POCUS image sequences. Preliminary results demonstrate the feasibility of training convolutional neural networks to distinguish physiologic from pathologic fluid collections, highlighting potential future applications for early detection of hemoperitoneum or ascites during emergency abdominal evaluation, particularly in resource-limited environments [43].

Earlier foundational studies further established the feasibility of applying artificial intelligence to abdominal ultrasound image analysis. Deep learning–based classification models achieved performance comparable to human readers for selected interpretive tasks. Although not specific to emergency care, these investigations introduced key technical principles—such as automated feature extraction from grayscale ultrasound images and tolerance to variable image quality—that continue to inform the development of contemporary AI-enhanced POCUS systems for acute abdominal assessment [44].

Key studies evaluating artificial intelligence–enhanced abdominal POCUS in non-traumatic emergencies, including clinical indications, AI methodologies, and reported outcomes, are summarized in Table 4.

### 4.4. Integrated Applications and Resource-Limited Settings

Beyond organ-specific applications, an expanding body of literature has examined AI-enhanced POCUS within integrated clinical workflows and in settings characterized by limited resources, workforce shortages, or restricted access to advanced imaging. In these contexts, artificial intelligence is primarily positioned as a means to support non-expert users, standardize image acquisition and interpretation, and extend the diagnostic reach of POCUS across diverse clinical environments.

Several studies highlight the particular relevance of AI-assisted POCUS in low- and middle-income countries and other resource-constrained or austere settings, where limited access to specialist expertise and diagnostic infrastructure remains a major barrier to timely care. In such environments, AI-supported ultrasound systems have been proposed as tools to enhance diagnostic confidence among frontline clinicians and to partially mitigate disparities in access to imaging-based decision support [46].

From a broader technological perspective, comprehensive reviews have explored the integration of artificial intelligence across ultrasound modalities, including POCUS, with emphasis on algorithm development, validation challenges, and barriers to clinical translation [47]. These analyses underscore that, while AI systems may demonstrate strong technical performance under controlled conditions, successful real-world deployment—particularly in low-resource settings—requires careful consideration of data bias, device heterogeneity, and regulatory oversight. Complementary engineering-focused studies further emphasize the importance of application-specific model design over generalized, one-size-fits-all approaches for ultrasound image analysis [48].

Practical implementations of AI-enhanced POCUS in integrated clinical scenarios have also been reported, describing multi-task frameworks capable of supporting image acquisition, quality assessment, and automated interpretation across multiple POCUS applications. Such integrated systems may offer greater clinical utility than isolated, single-task algorithms by aligning more closely with real-world workflow demands [9]. In parallel, lightweight AI models optimized for deployment on portable ultrasound devices have been developed, enabling point-of-care use in environments with limited computational resources [49]. Additional work has demonstrated the feasibility of real-time AI inference on edge devices, allowing ultrasound analysis without reliance on cloud connectivity [50].

Exploratory and preliminary reports further support these themes by describing AI-assisted ultrasound workflows for novice users and prototype systems that integrate AI feedback into procedural and perioperative ultrasound applications [51,52]. Earlier conceptual work anticipated these developments by proposing artificial intelligence as a means to standardize POCUS practice and expand its use beyond expert-dependent models, although empirical validation of such approaches remains limited [53].

More recent conceptual analyses have framed artificial intelligence as part of a broader evolution of emergency ultrasound, emphasizing that emerging technologies—including AI, cloud-based platforms, and augmented reality—should be meaningfully integrated into clinical reasoning processes rather than implemented as isolated technical solutions [1].

Key studies evaluating integrated AI-enhanced POCUS systems and applications in resource-limited or heterogeneous clinical settings, along with their technological approaches and clinical relevance, are summarized in Table 5.

### 4.5. Education and Training—AI-Enhanced POCUS Learning

Education and training are fundamental to the safe and effective use of point-of-care ultrasound (POCUS); however, traditional training models depend heavily on prolonged expert supervision, repeated hands-on practice, and subjective feedback. Achieving basic POCUS competency often requires a large number of supervised examinations, which can represent a significant barrier for learners, particularly in high-volume clinical environments or resource-limited settings [55].

Artificial intelligence has therefore emerged as a potential adjunct to support standardized instruction, provide objective feedback, and accelerate competency development across diverse clinical contexts.

Recent technological advances have enabled the development of AI-based systems capable of assessing image quality, guiding probe positioning, and delivering real-time corrective feedback during scanning [56].

By allowing trainees to identify and correct suboptimal technique at the bedside, these tools may reduce reliance on continuous expert supervision and improve the efficiency of skill acquisition.

Beyond acquisition support, AI-enhanced training approaches have demonstrated benefits in learning complex ultrasound views, particularly in cardiac POCUS. Real-time AI guidance has been associated with improved performance among novice users compared with conventional instruction alone, suggesting that automated feedback may accelerate proficiency in technically demanding applications [1].

Emerging evidence further supports the integration of AI into broader POCUS educational frameworks. AI-enabled handheld devices, simulation platforms, and structured training pathways that combine automated feedback with performance analytics offer scalable solutions to augment traditional teaching models, especially in settings with limited faculty availability [48]. Such approaches may facilitate individualized learning trajectories and enable longitudinal assessment of learner progression beyond isolated training sessions.

Importantly, the role of AI in POCUS education extends beyond image acquisition to interpretation skills. AI-assisted tools have shown potential to support novice users in identifying and classifying pathological findings during training, promoting the concurrent development of interpretative accuracy and technical competence [14].

Despite these opportunities, important limitations remain. The long-term effects of AI-based educational tools on skill retention, independent performance without AI assistance, and downstream clinical outcomes have yet to be fully established, underscoring the need for prospective and outcome-focused educational research [55].

Beyond instructional support, several recent studies highlight the potential of AI-based systems to provide objective and standardized assessment of POCUS competency. Machine learning–driven analysis of image quality, probe handling, and interpretative accuracy has been proposed as a means to reduce subjectivity in trainee evaluation and to support competency-based progression and credentialing. Such approaches may be particularly valuable in large training programs and resource-limited settings, where access to expert assessors is constrained and consistent benchmarking of performance remains challenging [57,58,59,60].

**Figure 1:**
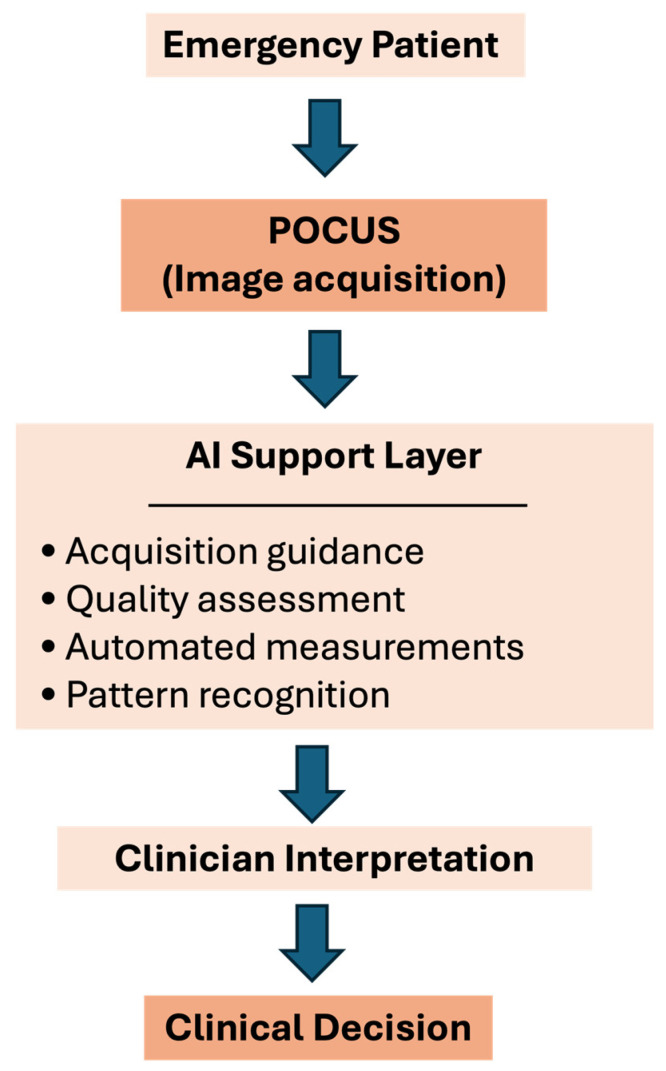
AI-enhanced POCUS workflow in emergency care.

Figure 1 schematic overview of the integration of artificial intelligence into point-of-care ultrasound (POCUS) workflows in emergency care. AI provides support for image acquisition, quality assessment, and interpretation, while final clinical decisions remain clinician-driven.

## 5. Limitations

Several limitations should be considered when interpreting the current evidence on AI-enhanced point-of-care ultrasound. Reproducibility remains a major concern, as many studies rely on retrospective designs, single-centre datasets, and predominantly internal validation, limiting generalizability to real-world emergency settings [61,62,63]. In addition, substantial heterogeneity in ultrasound devices, probes, software platforms, and acquisition protocols may significantly affect algorithm robustness and cross-device transferability [64].

The availability of large, diverse, and representative POCUS-specific datasets also remains limited, particularly in emergency, prehospital, and resource-limited environments, constraining robust external validation and increasing the risk of algorithmic bias [65].

Several authors caution that excessive reliance on automated image interpretation in the absence of appropriate clinician oversight may raise patient safety and medico-legal concerns. This underscores the need to frame AI-enhanced POCUS as a decision-support adjunct that complements, rather than substitutes for, clinician judgment [64,66].

A further limitation relates to the early developmental stage of much of the current AI–POCUS literature. A substantial proportion of published studies represent proof-of-concept or feasibility investigations conducted on curated datasets under controlled conditions. While these studies demonstrate technical potential, their findings should not be overinterpreted as indicators of clinical readiness or real-world effectiveness, particularly in heterogeneous emergency care environments. Moreover, although most AI–POCUS studies report performance metrics such as AUC, sensitivity, and accuracy, these measures alone do not adequately capture clinical utility [67]. The true impact of AI-assisted POCUS depends on whether algorithm outputs meaningfully support clinical decision-making or alter patient management. False-negative results may delay time-critical interventions, whereas false-positive findings may lead to unnecessary diagnostic procedures. To date, evidence that AI-enhanced POCUS consistently improves patient management or outcomes remains limited, as most studies prioritize technical validation over downstream clinical impact.

AI performance is also strongly influenced by acquisition conditions, including image quality, patient positioning, obesity-related acoustic limitations, and altered lung or cardiac mechanics during mechanical ventilation [2,20]. Because many models are trained and validated using data acquired under favorable conditions, their generalizability to real-world emergency settings may be limited.

Finally, ultrasound device heterogeneity represents an important methodological constraint. Most AI–POCUS systems are developed and validated on single-vendor platforms or rely on vendor-specific, closed algorithms [55]. Given that emergency departments routinely employ devices from multiple manufacturers with differing hardware characteristics and image processing pipelines, performance estimates derived from single-vendor data may not translate reliably across platforms. Evidence regarding cross-vendor robustness remains sparse, underscoring the need for vendor-agnostic model development, standardized data formats, and prospective multicentre validation.

## 6. Conclusions

Artificial intelligence is increasingly influencing the evolution of point-of-care ultrasound in emergency medicine by addressing challenges related to operator dependency, interpretive variability, and time-critical decision-making. The evidence summarized in this review indicates that AI-enhanced POCUS can support multiple stages of the ultrasound workflow, including image acquisition, quality assessment, automated quantification, and interpretation, across both traumatic and non-traumatic emergency settings.

In trauma care, AI-assisted POCUS shows promise for automated detection of pneumothorax, hemothorax, and free intraperitoneal fluid, with potential to standardize eFAST examinations and support rapid triage. In non-traumatic emergencies, cardiovascular, pulmonary, and abdominal applications suggest that AI can provide quantitative and pattern-recognition support—such as ejection fraction estimation and lung ultrasound analysis—particularly when image quality is adequate and clinical use cases are well defined.

Integrated AI-POCUS systems, applications in resource-limited settings, and AI-supported educational tools further highlight the potential of artificial intelligence to expand access to ultrasound, assist non-expert users, and reduce variability in training and skill acquisition.

Nevertheless, much of the current evidence remains early-stage, and reported performance metrics do not consistently translate into demonstrated clinical impact. Model reliability is strongly influenced by acquisition conditions, dataset characteristics, and device heterogeneity, while limited cross-vendor validation constrains generalizability. These findings reinforce the role of AI-enhanced POCUS as a decision-support technology that augments, rather than replaces, clinician judgment.

Overall, AI-enhanced POCUS is progressing toward early clinical integration, but its safe and effective adoption will require prospective multicentre validation, larger and more representative datasets, vendor-agnostic model development, and alignment with clinical, ethical, and regulatory frameworks.

## Figures and Tables

**Table 1:** AI-assisted POCUS applications for trauma assessment in emergency care.

Setting/Focus	AI Approach	Key Findings	Sample Size (Approx.)	Validation Type	Dataset Origin	References
Emergency eFAST	Real-time anatomical landmark detection + classifier	Combined image guidance and interpretation demonstrated feasibility in emergency triage.	200–400 exams	Internal	Single-centre	[13]
Lung ultrasound pneumothorax	Stepwise DL for QA and sliding classification	AUC ~0.89 for full pipeline; high reliability for diagnostic support.	800–1200 video clips	External	Multi-centre	[14]
Pneumothorax detection	Neural network on POCUS	Sensitivity ~86% for PTX detection, illustrating potential clinical performance.	500–1000 images	Internal	Multi-centre	[15]
Thoracic trauma (swine model)	CNN classification (MobileNetV3)	Real-time M-mode PTX/HTX detection with ~85% accuracy; reduces required expertise threshold.	300–500 M-mode clips	Internal	Single-centre	[16]
Lung trauma (pneumothorax)	CNN-based deep learning	Automated pneumothorax detection with high diagnostic accuracy	500–1000 lung US frames/clips	Internal	Single-centre	[17]
Lung trauma (pneumothorax)	Deep learning (CNN)	Automated pneumothorax detection on lung ultrasound with strong diagnostic performance	400–800 lung US images/clips	Internal	Single-centre	[18]

**Table 2:** Summary of AI-assisted POCUS applications for cardiovascular assessment in non-traumatic emergencies.

Setting/Population	AI Task	Key Findings	Sample Size (Approx.)	Validation Type	Dataset Origin	References
ED/ICU patients; cardiac POCUS clips	On-device AI (auto-EF, auto-VTI, auto-IVC)	Moderate–good agreement with expert POCUS for high-quality views (κ ≈ 0.50–0.66)	200–400 clips	Internal	Single-centre	[19]
Emergency department adults ≥45 years	Vendor AI for systolic/diastolic dysfunction	Sensitivity 85–92%; specificity 94–95% vs. expert reviewers	~200 patients	External	Single-centre	[26]
Unstable ED/ICU patients; PLAX POCUS	CNN-based wall-tracking	Accurate EF classification (85–87%) from parasternal long-axis view	500–700 studies	Internal	Multi-centre	[27]
ED and community cardiac POCUS	CNN screening for cardiomyopathies	AUROC~0.90–0.97; early detection of HCM and ATTR-CM	>40,000 videos	External	Multi-centre	[21]
Emergency department cardiac POCUS	Deep learning (EchoNet-Dynamic)	Reduced performance on POCUS vs. formal echo (Dice~0.72; κ~0.16)	300–400 videos	External	Single-centre	[23]
Subxiphoid cardiac POCUS views	Machine learning with data augmentation	Feasible EF estimation; higher error at mid-range EF values	500–700 clips	Internal	Single-centre	[24]
Neonatal non-traumatic emergencies	ML/DL-assisted targeted echocardiography	Early-stage and conceptual applications for bedside hemodynamic assessment	Not specified	Narrative/Conceptual	Multi-centre	[22]
Cardiovascular POCUS platforms	Integrated AI quantification tools (AutoEF, SmartVTI)	Demonstrated technical maturity and clinical feasibility in acute care	Not specified	Narrative/Technology overview	Multi-centre	[25]
Cardiac ultrasound (LV function)	Machine learning–based EF estimation	Feasible automated estimation of left ventricular function from ultrasound images	100–300 studies	Internal	Single-centre	[28]
Perioperative and critical care cardiac POCUS	AI-assisted cardiac function assessment	AI-supported quantification of cardiac function feasible and clinically relevant in acute care settings	150–300 examinations	Internal	Single-centre	[29]

**Table 3:** AI-assisted lung POCUS in non-traumatic emergencies.

Clinical Context	Lung POCUS Application	AI Approach	Main Findings	Sample Size (Approx.)	Validation Type	Dataset Origin	References
Emergency department; acute dyspnea	Detection of B-lines and pleural abnormalities	CNN-based image classification	Good discrimination between normal and abnormal lung patterns (preliminary)	Not specified	Not specified	Not specified	[34]
Suspected pneumothorax	Pleural sliding analysis; pneumothorax detection	Deep learning on annotated ultrasound video loops	High diagnostic performance (AUC > 0.90) vs. expert interpretation	Several hundred-1000 clips (reported as large dataset)	Internal (development/validation)	Single-centre/not clearly stated	[30]
Acute dyspnea/heart failure	Automated B-line quantification	Machine learning–based feature extraction and classification	Strong correlation with expert annotations and congestion biomarkers	Not specified	Not specified	Not specified	[31]
Pleural disease evaluation	Differentiation of pleural effusion types	Pattern recognition algorithms	Improved accuracy in pleural fluid characterization	Not specified	Internal	Single-centre	[32]
Emergency lung ultrasound workflows	Image acquisition support and standardization	Prototype AI-guided acquisition system	Feasibility of AI feedback for improving scan quality	Not specified	Prototype/feasibility	Not specified	[33]
Lung ultrasound data development	Lung POCUS image labeling for AI training	Crowdsourcing-assisted annotation with ML support	Demonstrated feasibility of scalable, high-quality annotation for lung ultrasound datasets	Large annotated dataset (exact size reported in study)	Not applicable (data development)	Multi-centre/crowdsourced	[35]
Acute care/emergency lung ultrasound	Automated lung ultrasound pattern recognition	Deep learning (CNN-based)	Demonstrated feasibility of AI-assisted lung pattern classification on POCUS images	Several hundred images/clips	Internal	Single-centre	[36]
Lung ultrasound (acute and emergency care)	Automated lung ultrasound image analysis	Deep learning (CNN-based)	Demonstrated feasibility of AI-based lung ultrasound pattern recognition	Tens to low hundreds of images/clips	Internal	Single-centre	[37]
Emergency and acute care lung ultrasound	Automated lung ultrasound interpretation	Deep learning (CNN-based)	Demonstrated feasibility of AI-assisted lung ultrasound analysis with clinically relevant performance	Several hundred lung US images/clips	Internal	Single-centre	[38]
Acute and emergency lung ultrasound	Automated lung ultrasound pattern classification	Deep learning (CNN-based)	AI model achieved reliable lung pattern classification on POCUS images	Several hundred lung US images/clips	Internal	Single-centre	[39]
Lung ultrasound image analysis	Automated lung ultrasound feature and pattern detection	Deep learning (CNN-based)	Demonstrated feasibility of automated lung ultrasound image analysis with promising classification performance	Several hundred images	Internal	Single-centre	[40]
Lung ultrasound image analysis	Automated lung ultrasound pattern classification	Deep learning (CNN-based)	Demonstrated accurate automated classification of lung ultrasound patterns under controlled conditions	Retrospective analysis; not specific to emergency workflows	Several hundred images/clips	Internal	[41]

**Table 4:** AI-assisted abdominal POCUS in non-traumatic emergencies.

Clinical Context	Abdominal Application	AI Approach	Main Findings	Sample Size (Approx.)	Validation Type	Dataset Origin	References
Obstetric and gynecologic emergencies	Early pregnancy assessment; risk stratification	Machine learning–based image interpretation	AI-assisted POCUS supported rapid differentiation of intrauterine vs. ectopic pregnancy	Not specified	Internal	Single-centre	[42]
Acute abdominal evaluation	Detection of free intraperitoneal fluid	CNN-based image and video classification	Demonstrated feasibility of automated free fluid detection	Not specified	Not specified	Not specified	[43]
General abdominal ultrasound	Automated image classification	Deep learning models	Performance comparable to human readers for selected tasks	Several hundred images	Internal	Single-centre	[44]
Abdominal ultrasound image analysis	Automated abdominal organ and pathology classification	Deep learning (CNN-based)	Demonstrated accurate automated classification of abdominal ultrasound images	Several hundred images	Internal	Single-centre/curated dataset	[45]

**Table 5:** Integrated and resource-oriented applications of AI-assisted POCUS.

Setting/Focus	AI Application	AI Approach	Key Contribution	Sample Size	Validation Type	Dataset Origin	References
Low- and middle-income countries (LMICs)	AI-assisted diagnostic POCUS	ML/DL-based image interpretation	Demonstrated feasibility and relevance in constrained healthcare environments	Not specified	Narrative/feasibility	Multi-centre/heterogeneous	[46]
Cross-modality ultrasound	Translational AI in ultrasound imaging	Review of ML/DL architectures	Identified barriers to clinical translation	Not applicable	Narrative review	Multi-centre	[47]
Engineering-focused AI for POCUS	Application-specific DL model design	Deep learning architectures	Highlighted importance of task-specific AI models for robust POCUS deployment	Not applicable	Engineering/methodological	Single-/Multi-centre datasets	[48]
Integrated POCUS workflows	Multi-task AI systems	CNN-based pipelines	Demonstrated feasibility of integrated AI support across the POCUS workflow	Not specified	Prototype/feasibility	Single-centre	[9]
Portable devices	Lightweight AI models for edge deployment	Optimized DL models	Optimized for low-compute environments	Not specified	Technical/feasibility	Single-centre	[49]
Trauma and acute care workflows	AI-assisted POCUS for trauma assessment	Deep learning–based image interpretation and decision support	Demonstrated feasibility of AI-supported ultrasound interpretation to assist trauma evaluation and triage	Several hundred examinations	Internal	Single-centre	[54]
System-wide emergency ultrasound practice	AI-assisted POCUS adoption and implementation	Survey-based evaluation of ML-enabled POCUS tools	Identified key clinical, technical, and organizational barriers to AI-POCUS adoption (training, trust, workflow integration)	Several hundred clinicians	Observational survey	Multi-centre/international	[10]

## Data Availability

No new data were created or analyzed in this study. Data sharing is not applicable to this article.

## References

[1] Osterwalder J., Polyzogopoulou E., Hoffmann B. (2023). Point-of-Care Ultrasound—History, Current and Evolving Clinical Concepts in Emergency Medicine. Medicina.

[2] Kim J., Maranna S., Watson C., Parange N. (2025). A scoping review on the integration of artificial intelligence in point-of-care ultrasound: Current clinical applications. Am. J. Emerg. Med..

[3] East S.A., Wang Y., Yanamala N., Maganti K., Sengupta P.P. (2025). Artificial Intelligence-Enabled Point-of-Care Echocardiography: Bringing Precision Imaging to the Bedside. Curr. Atheroscler. Rep..

[4] Yordanova M.Z. (2024). The Applications of Artificial Intelligence in Radiology: Opportunities and Challenges. Eur. J. Med. Health Sci..

[5] Yan L., Li Q., Fu K., Zhou X., Zhang K. (2025). Progress in the Application of Artificial Intelligence in Ultrasound-Assisted Medical Diagnosis. Bioengineering.

[6] He L., Luan L., Hu D. (2025). Deep learning-based image classification for AI-assisted integration of pathology and radiology in medical imaging. Front. Med..

[7] Zhang X.M., Gao T.H., Cai Q.Y., Xia J.B., Sun Y.N., Yang J., Li W.-H., Zhang S.-X., Lou H.-R., Yu X.-T. (2026). Artificial intelligence in digital pathology diagnosis and analysis: Technologies, challenges, and future prospects. Mil. Med. Res..

[8] Myhre P.L., Grenne B., Asch F.M., Delgado V., Khera R., Lafitte S., Lang R.M., Pellikka P.A., Sengupta P.P., Vemulapalli S. (2025). Artificial intelligence-enhanced echocardiography in cardiovascular disease management. Nat. Rev. Cardiol..

[9] Kim S., Fischetti C., Guy M., Hsu E., Fox J., Young S.D. (2024). Artificial Intelligence (AI) Applications for Point of Care Ultrasound (POCUS) in Low-Resource Settings: A Scoping Review. Diagnostics.

[10] Wong A., Roslan N.L., McDonald R., Noor J., Hutchings S., D’Costa P., Via G., Corradi F. (2025). Clinical obstacles to machine-learning POCUS adoption and system-wide AI implementation (The COMPASS-AI survey). Ultrasound J..

[11] Koçak B., Ponsiglione A., Stanzione A., Bluethgen C., Santinha J., Ugga L., Huisman M., Klontzas M.E., Cannella R., Cuocolo R. (2024). Bias in artificial intelligence for medical imaging: Fundamentals, detection, avoidance, mitigation, challenges, ethics, and prospects. Diagn. Interv. Radiol..

[12] Osifowokan A.S., Agbadamasi T.O., Adukpo T.K., Mensah N. (2025). Regulatory and legal challenges of Artificial Intelligence in the U.S. Healthcare System: Liability, Compliance, and Patient Safety. World J. Adv. Res. Rev..

[13] Hernandez Torres S.I., Holland L., Winter T., Ortiz R., Amezcua K.L., Ruiz A., Thorpe C.R., Snider E.J. (2025). Real-Time Deployment of Ultrasound Image Interpretation AI Models for Emergency Medicine Triage Using a Swine Model. Technologies.

[14] Kim K., Macruz F., Wu D., Bridge C., McKinney S., Al Saud A.A., Sharaf E., Sesic I., Pely A., Danset P. (2023). Point-of-care AI-assisted stepwise ultrasound pneumothorax diagnosis. Phys. Med. Biol..

[15] Montgomery S., Li F., Funk C., Peethumangsin E., Morris M., Anderson J.T., Hersh A.M., Aylward S. (2023). Detection of pneumothorax on ultrasound using artificial intelligence. J. Trauma. Acute Care Surg..

[16] Ruiz A.J., Hernández Torres S.I., Snider E.J. (2025). Development of Deep Learning Models for Real-Time Thoracic Ultrasound Image Interpretation. J. Imaging.

[17] Yıldız Potter İ., Leo M.M., Vaziri A., Feldman J.A. (2023). Automated detection and localization of pericardial effusion from point-of-care cardiac ultrasound examination. Med. Biol. Eng. Comput..

[18] Leo M.M., Potter I.Y., Zahiri M., Vaziri A., Jung C.F., Feldman J.A. (2023). Using Deep Learning to Detect the Presence and Location of Hemoperitoneum on the Focused Assessment with Sonography in Trauma (FAST) Examination in Adults. J. Digit. Imaging.

[19] Gohar E., Herling A., Mazuz M., Tsaban G., Gat T., Kobal S., Fuchs L. (2023). Artificial Intelligence (AI) versus POCUS Expert: A Validation Study of Three Automatic AI-Based, Real-Time, Hemodynamic Echocardiographic Assessment Tools. J. Clin. Med..

[20] Mika S., Gola W., Gil-Mika M., Wilk M., Misiołek H. (2024). Overview of artificial intelligence in point-of-care ultrasound. New horizons for respiratory system diagnoses. Anaesthesiol. Intensive Ther..

[21] Oikonomou E.K., Vaid A., Holste G., Coppi A., McNamara R.L., Baloescu C., Krumholz H.M., Wang Z., Apakama D.J., Nadkarni G.N. (2025). Artificial intelligence-guided detection of under-recognised cardiomyopathies on point-of-care cardiac ultrasonography: A multicentre study. Lancet Digit. Health.

[22] Singh Y. (2024). Echocardiography in the neonatal unit: Current status and future prospects. Expert Rev. Med. Devices.

[23] Crockett D., Kelly C., Brundage J., Jones J., Ockerse P. (2022). A Stress Test of Artificial Intelligence: Can Deep Learning Models Trained From Formal Echocardiography Accurately Interpret Point-of-Care Ultrasound?. J. Ultrasound Med..

[24] Blaivas M., Blaivas L.N., Campbell K., Thomas J., Shah S., Yadav K., Liu Y.T. (2022). Making Artificial Intelligence Lemonade Out of Data Lemons: Adaptation of a Public Apical Echo Database for Creation of a Subxiphoid Visual Estimation Automatic Ejection Fraction Machine Learning Algorithm. J. Ultrasound Med..

[25] Vega R., Dehghan M., Nagdev A., Buchanan B., Kapur J., Jaremko J.L., Zonoobi D. (2025). Overcoming barriers in the use of artificial intelligence in point of care ultrasound. npj Digit. Med..

[26] Gottlieb M., Schraft E., O’Brien J., Patel D. (2024). Diagnostic accuracy of artificial intelligence for identifying systolic and diastolic cardiac dysfunction in the emergency department. Am. J. Emerg. Med..

[27] Vega R., Nagdev A., Dehghan M., Seyed Bolouri S.E., Buchanan B., Kapur J., Jaremko J.L., Zonoobi D. (2025). A wall tracking method to estimate ejection fraction from the parasternal long axis view in point of care ultrasound. WFUMB Ultrasound Open.

[28] Blaivas M., Blaivas L.N., Tsung J.W. (2021). Deep learning algorithm performance compared to experts in visual evaluation of interior vena cava collapse on ultrasound to determine intravenous fluid need in dehydration management. Signa Vitae.

[29] Efrimescu C.I., Moorthy A., Griffin M. (2023). Rescue Transesophageal Echocardiography: A Narrative Review of Current Knowledge and Practice. J. Cardiothorac. Vasc. Anesth..

[30] Ienghong K., Cheung L.W., Gaysonsiri D., Apiratwarakul K. (2025). The diagnostic performance of automatic B-lines detection for evaluating pulmonary edema in the emergency department among novice point-of-care ultrasound practitioners. Emerg. Radiol..

[31] Duggan N.M., Jin M., Duran Mendicuti M.A., Hallisey S., Bernier D., Selame L.A., Asgari-Targhi A., Fischetti C.E., Lucassen R., Samir A.E. (2024). Gamified Crowdsourcing as a Novel Approach to Lung Ultrasound Data Set Labeling: Prospective Analysis. J. Med. Internet Res..

[32] Nti B., Lehmann A.S., Haddad A., Kennedy S.K., Russell F.M. (2022). Artificial Intelligence-Augmented Pediatric Lung POCUS: A Pilot Study of Novice Learners. J. Ultrasound Med..

[33] Moore C.L., Wang J., Battisti A.J., Chen A., Fincke J., Wang A., Wagner M., Raju B., Baloescu C. (2022). Interobserver Agreement and Correlation of an Automated Algorithm for B-Line Identification and Quantification with Expert Sonologist Review in a Handheld Ultrasound Device. J. Ultrasound Med..

[34] Jeffers K., Keim S.M., Long B., Gottlieb M., Adhikari S.R. (2025). What is the Utility of Point-of-Care Ultrasound for Diagnosing Pulmonary Edema?. J. Emerg. Med..

[35] Song F., Liu H., Ma H., Chen X., Wang S., Qin T., Liang H., Huang D. (2025). AI Model Based on Diaphragm Ultrasound to Improve the Predictive Performance of Invasive Mechanical Ventilation Weaning: Prospective Cohort Study. JMIR Form Res.

[36] Labaf A., Åhman-Persson L., Husu L.S., Smith J.G., Ingvarsson A., Evaldsson A.W. (2025). Performance of a point-of-care ultrasound platform for artificial intelligence-enabled assessment of pulmonary B-lines. Cardiovasc. Ultrasound.

[37] Sultan L.R., Haertter A., Al-Hasani M., Demiris G., Cary T.W., Tung-Chen Y., Sehgal C.M. (2023). Can Artificial Intelligence Aid Diagnosis by Teleguided Point-of-Care Ultrasound? A Pilot Study for Evaluating a Novel Computer Algorithm for COVID-19 Diagnosis Using Lung Ultrasound. AI.

[38] Schneider E., Maimon N., Hasidim A., Shnaider A., Migliozzi G., Haviv Y.S., Halpern D., Abu Ganem B., Fuchs L. (2023). Can Dialysis Patients Identify and Diagnose Pulmonary Congestion Using Self-Lung Ultrasound?. J. Clin. Med..

[39] Kuroda Y., Kaneko T., Yoshikawa H., Uchiyama S., Nagata Y., Matsushita Y., Hiki M., Minamino T., Takahashi K., Daida H. (2023). Artificial intelligence-based point-of-care lung ultrasound for screening COVID-19 pneumoniae: Comparison with CT scans. Valera-Calero JA, editor. PLoS ONE.

[40] Song J., Ebadi A., Florea A., Xi P., Tremblay S., Wong A. (2023). COVID-Net USPro: An Explainable Few-Shot Deep Prototypical Network for COVID-19 Screening Using Point-of-Care Ultrasound. Sensors.

[41] Baloescu C., Toporek G., Kim S., McNamara K., Liu R., Shaw M.M., McNamara R.L., Raju B.I., Moore C.L. (2020). Automated Lung Ultrasound B-Line Assessment Using a Deep Learning Algorithm. IEEE Trans. Ultrason. Ferroelectr. Freq. Control.

[42] Mathyk B., Pandya S., Wright Beatty H., Anderson M.L., Kohut A. (2025). Handheld Point-of-Care Ultrasonography for Gynecology: Insights Into Space Travel Through Parabolic Flight. Obstet. Gynecol..

[43] Zgool T., Antico M., Edwards C., Fontanarosa D. (2025). Point-of-Care Ultrasound Imaging for Automated Detection of Abdominal Haemorrhage: A Systematic Review. Ultrasound Med. Biol..

[44] Lee K., Kim M., Lim C., Song T.K. (2021). Reverse Scan Conversion and Efficient Deep Learning Network Architecture for Ultrasound Imaging on a Mobile Device. Sensors.

[45] Yu C.J., Yeh H.J., Chang C.C., Tang J.H., Kao W.Y., Chen W.C., Huang Y.-J., Li C.-H., Chang W.-H., Lin Y.-T. (2021). Lightweight deep neural networks for cholelithiasis and cholecystitis detection by point-of-care ultrasound. Comput. Methods Programs Biomed..

[46] Zahid M.A., Nasir H., Abid S., Shuja Ul Islam M.H.S., Sawwa A., Jamil S. (2025). Why is it necessary for every medical specialty to learn point-of-care ultrasound (POCUS)? A comprehensive review. Anaesth. Pain. Intensive Care.

[47] Kaffas A.E., Vo-Phamhi J.M., Griffin J.F., Hoyt K. (2024). Critical Advances for Democratizing Ultrasound Diagnostics in Human and Veterinary Medicine. Annu. Rev. Biomed. Eng..

[48] Venkatayogi N., Gupta M., Gupta A., Nallaparaju S., Cheemalamarri N., Gilari K., Pathak S., Vishwanath K., Soney C., Bhattacharya T. (2023). From Seeing to Knowing with Artificial Intelligence: A Scoping Review of Point-of-Care Ultrasound in Low-Resource Settings. Appl. Sci..

[49] Alfoti B.O.O., Alfoti F.O.O., Alothman S.T.H., Al-Dhafiri T.M.A., Al-Harbi N.H.M., Al-Khalidi A.M., Alzahrany N.D.B., Abutalib F.M., Almojam S.A., Ali M.A.-H.S. (2024). Utilization of Point-of-Care Ultrasound (POCUS) in Emergency and Critical Care: Role of Nursing for Enhancing Diagnostic Accuracy and Efficiency-Systematic Review. Egypt. J. Chem..

[50] Zeng E.Z., Ebadi A., Florea A., Wong A. (2024). COVID-Net L2C-ULTRA: An Explainable Linear-Convex Ultrasound Augmentation Learning Framework to Improve COVID-19 Assessment and Monitoring. Sensors.

[51] Yazici M.M., Yavaşi Ö. (2025). The development of point-of-care ultrasound (POCUS): Worldwide contributions and publication trends. J. Clin. Ultrasound.

[52] Park Y., Han J., Leikin S., Díaz-Gómez J.L. (2024). Essential Point-of-Care Ultrasound Insights for 2024. Semin. Ultrasound CT MRI.

[53] Blaivas M., Arntfield R., White M. (2020). Creation and Testing of a Deep Learning Algorithm to Automatically Identify and Label Vessels, Nerves, Tendons, and Bones on Cross-sectional Point-of-Care Ultrasound Scans for Peripheral Intravenous Catheter Placement by Novices. J. Ultrasound Med..

[54] Rowe M., Ferrada P. (2025). Ultrasound to guide critical decisions: All that you need to know. J. Trauma Acute Care Surg..

[55] Blaivas M., Arntfield R., White M. (2020). DIY AI, deep learning network development for automated image classification in a point-of-care ultrasound quality assurance program. J. Am. Coll. Emerg. Physicians Open.

[56] Chou H.H., Chang Y.C., Lien W.C., Lin L.C., Lin X.Z., Hsu T.E., Liu Y.-P., Liu L., Chan Y.-T., Kuan F.-S. (2025). Efficient Deep Learning Models Revolutionize Doctor’s Training for Point-of-Care Ultrasound. IEEE Access.

[57] Karni O., Shitrit I.B., Perlin A., Jedwab R., Wacht O., Fuchs L. (2025). AI-enhanced guidance demonstrated improvement in novices’ Apical-4-chamber and Apical-5-chamber views. BMC Med. Educ..

[58] Aronovitz N., Hazan I., Jedwab R., Ben Shitrit I., Quinn A., Wacht O., Fuchs L. (2024). The effect of real-time EF automatic tool on cardiac ultrasound performance among medical students. AbdelMassih AF, editor. PLoS ONE.

[59] Dadon Z., Orlev A., Butnaru A., Rosenmann D., Glikson M., Gottlieb S., Alpert E.A. (2023). Empowering Medical Students: Harnessing Artificial Intelligence for Precision Point-of-Care Echocardiography Assessment of Left Ventricular Ejection Fraction. Int. J. Clin. Pract..

[60] Zhai S., Wang H., Sun L., Zhang B., Huo F., Qiu S., Wu X., Ma J., Wu Y., Duan J. (2022). Artificial intelligence (AI) versus expert: A comparison of left ventricular outflow tract velocity time integral (LVOT-VTI) assessment between ICU doctors and an AI tool. J. Appl. Clin. Med. Phys..

[61] Lin-Martore M., Kornblith A., Firnberg M., Haque A., O’Brien B. (2025). Trust of Artificial Intelligence-Augmented Point-of-Care Ultrasound Among Pediatric Emergency Physicians. J. Am. Coll. Emerg. Physicians Open.

[62] Shokoohi H., Liteplo A.S., Montoya K., Patnode C., Hutchinson A.B., Zalis M.E., Gottlieb M., Raja A.S., Slutzman J.E. (2025). Climate-Smart Diagnostic Medical Imaging and Point-of-Care Ultrasound: An Evidence-Based Perspective. J. Emerg. Med..

[63] Kayarian F., Patel D., O’Brien J.R., Schraft E.K., Gottlieb M. (2024). Artificial intelligence and point-of-care ultrasound: Benefits, limitations, and implications for the future. Am. J. Emerg. Med..

[64] Blaivas M., Blaivas L.N., Tsung J.W. (2022). Deep Learning Pitfall: Impact of Novel Ultrasound Equipment Introduction on Algorithm Performance and the Realities of Domain Adaptation. J. Ultrasound Med..

[65] Suttels V., Du Toit J.D., Fiogbé A.A., Wachinou A.P., Guendehou B., Alovokpinhou F., Toukoui P., Hada A.R., Sefou F., Vinasse P. (2022). Point-of-care ultrasound for tuberculosis management in Sub-Saharan Africa—A balanced SWOT analysis. Int. J. Infect. Dis..

[66] Blaivas L., Blaivas M. (2021). Are Convolutional Neural Networks Trained on ImageNet Images Wearing Rose-Colored Glasses?: A Quantitative Comparison of IMAGENET, Computed Tomographic, Magnetic Resonance, Chest X-Ray, and Point-of-Care Ultrasound Images for Quality. J. Ultrasound Med..

[67] Kameda T., Ishii H., Oya S., Katabami K., Kodama T., Sera M., Takei H., Taniguchi H., Nakao S., Funakoshi H. (2024). Guidance for clinical practice using emergency and point-of-care ultrasonography. Acute Med. Surg..

